# Gastric Siderosis and Ulceration from Intravenous Iron Supplementation Manifesting as Chronic Upper Gastrointestinal Bleeding: A Case Report and Review of the Literature

**DOI:** 10.1155/2019/1790686

**Published:** 2019-04-18

**Authors:** Donald Ewing, Ava Brozovich, Ethan Burns, Gonzalo Acosta, Courtney Hatcher, Pragnesh Patel, Rose Anton, Bincy Abraham, Leena Samuel

**Affiliations:** ^1^Texas A&M University College of Medicine, 8447 Bryan Rd., Bryan, TX 77807, USA; ^2^Houston Methodist Hospital, Department of Medicine, 6550 Fannin St., Houston, TX 77096, USA; ^3^Houston Methodist Hospital, Department of Gastroenterology, 6550 Fannin St., Houston, TX 77096, USA; ^4^Houston Methodist Hospital, Department of Pathology and Genomic Medicine, 6550 Fannin St., Houston, TX 77030, USA

## Abstract

Iron deficiency is the most common etiology of anemia worldwide and is often managed with varying methods of iron supplementation. Although rare, oral iron supplementation can perpetuate iron deficiency anemia by causing gastric ulceration and upper gastrointestinal bleeding in high-risk populations. However, this complication has not been previously described with intravenous iron supplementation. We present a case of a 63-year-old male with severe iron deficiency anemia on biweekly intravenous iron infusions and weekly packed red blood cell transfusions who presented with melena over several months. Upper endoscopy demonstrated a clean-based gastric body ulcer and nonbleeding gastric varices. Histology of the gastric ulcer was suggestive of iron-induced gastric mucosal injury. This case demonstrates that frequent utilization of intravenous iron and packed red blood cell transfusions may predispose certain patients to the development of iron-induced gastritis and ulceration.

## 1. Introduction

The prevalence of anemia in the global population is estimated to be 24.8%, with iron deficiency as the most common etiology [1]. Oral iron supplementation is the most conventional treatment modality for iron deficiency anemia (IDA) with intravenous (IV) iron and packed red blood cell (PRBC) transfusions reserved for specific circumstances such as oral iron intolerance, history of gastric surgery, chronic blood loss anemia, malabsorptive syndromes, and severe deficiency/anemia [2]. Adverse effects of oral iron therapy may include a metallic taste, nausea, constipation, diarrhea, and flatulence; nonetheless it is generally well tolerated [2]. Uncommon but potentially serious adverse effects are chronic gastritis, ulceration, and bleeding secondary to oxidative damage to the gastric mucosa [3–5].

Iron-induced gastritis is a rare condition that is scarcely reported in the literature. We present a unique case of iron-induced gastritis due to recurrent PRBC transfusions and IV iron infusions.

## 2. Presentation of Case

A 69-year-old Caucasian male with history of a precancerous supraglottic mass treated with resection and radiation, compensated alcoholic cirrhosis, and large ascending colon polyp treated with right hemicolectomy 4-years-ago presented with melena for the past 4 months. The patient denied nausea, vomiting, abdominal pain, hematemesis, hematochezia, reflux symptoms, change in bowel habits, or weight loss. He denied use of nonsteroidal anti-inflammatory agents.

The patient had episodes of intermittent melena for the past 3 years requiring blood transfusions, but it had become a daily occurrence in the past 4 months. Prior to presenting for this current admission, the patient had two esophagogastroduodenoscopies (EGD), two colonoscopies, and one video capsule endoscopy that failed to identify a source of his melena. In the last 2 months, his hemoglobin has ranged between 6.2 g/dL and 7.4 g/dL requiring 2 units of PRBCs weekly as well as biweekly IV iron infusions. He had not used oral iron supplementation in the 6 months prior to admission.

**Figure 1:**
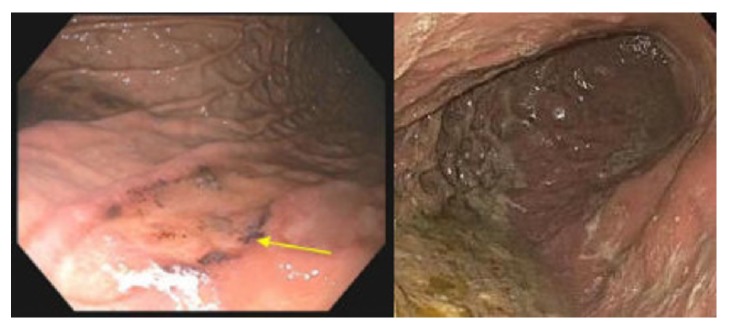
(a) EGD at admission. Nonbleeding ulcer located in the gastric body. There is black mucosal discoloration present. Biopsies were taken. (b) Gastric body during repeated EGD 6 months later. Significant residue in the stomach. Ulceration not visualized. EGD: esophagogastroduodenoscopy.

**Figure 2:**
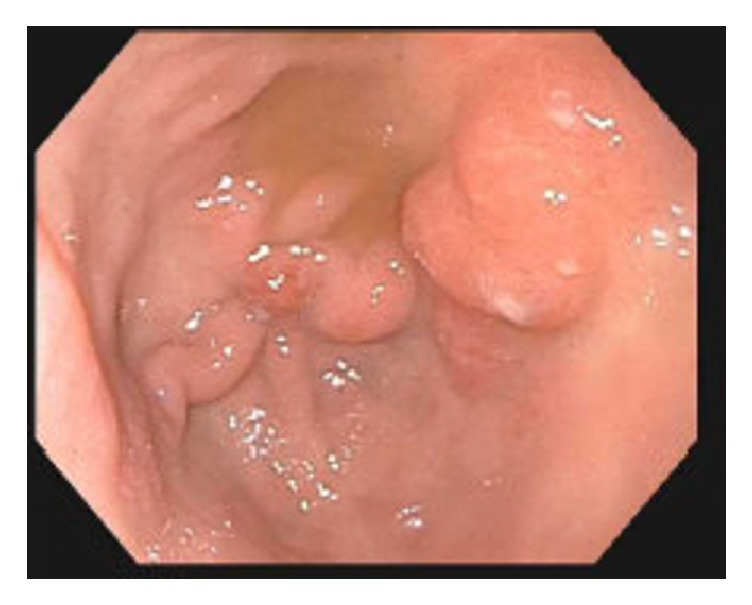
Nonbleeding gastric varices.

**Figure 3:**
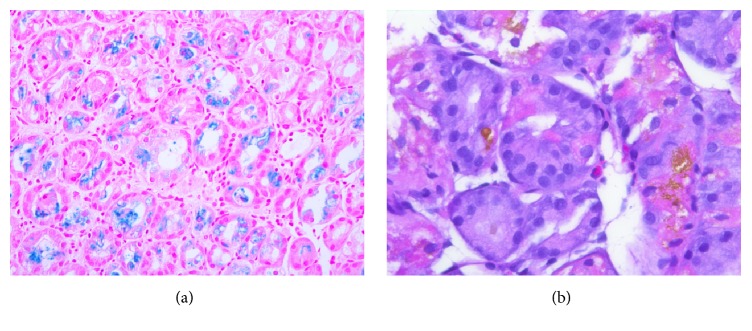
(a) An iron stain with intraepithelial and intraluminal iron (blue stain). Prussian blue, 200x. (b) A high-power view of the stomach shows parietal and chief cells containing intracytoplasmic yellow-orange coarse granules consistent with iron deposition. H&E, 400x.

On presentation, the patient was asymptomatic and hemodynamically stable. Rectal exam revealed black, tarry stool in the rectal vault without hemorrhoids or a palpable rectal mass. Blood work was significant for a hemoglobin of 4.4 g/dL and acute kidney injury, for which he received two units of PRBCs. His ferritin was 109 ng/mL, transferrin 194 mg/dL, TIBC 225 ug/dL, iron level 38 ug/dL, and percent iron saturation 16.9%, supporting the diagnosis of IDA. Computed Tomography (CT) of the abdomen and pelvis was notable for cirrhosis. An esophagogastroduodenoscopy (EGD) demonstrated a nonbleeding clean-based ulcer in the gastric body (Figure 1(a)) and nonbleeding gastric varices (Figure 2). Biopsies indicated heavy iron deposition, and immunostaining for* Helicobacter pylori* (H. Pylori) was negative (Figure 3). Iron therapy was discontinued and treatment with a proton pump inhibitor was initiated. The patient's hemoglobin remained stable and he was discharged. On follow-up, the patient's melena had resolved and after 9 months his hemoglobin was stable at 11.2g/dL. Repeated EGD did not locate an ulcer (Figure 1(b)), and histology showed chronic inactive gastritis. Repeated iron staining was not performed.

## 3. Discussion

According to a population-based study of 3,000 participants in Sweden, the prevalence of peptic ulcer disease (PUD) is approximately 4.1% (gastric=2.0%, duodenal=2.1%) [6, 7], which has decreased over time with treatment of *H*. pylori [7] infections and gastric acid suppression with proton pump inhibitors [8]. In developed countries, the frequent use of aspirin and nonsteroidal anti-inflammatory drugs has become a more common cause of PUD [7]. Iron-induced gastric ulcers are a rare phenomenon and are previously reported in association with oral iron pill therapy [3–5]. To our knowledge, there are no reported cases in the literature demonstrating gastritis or gastric ulceration due to intravenous iron or PRBC transfusions.

Iron deposition in the gastric mucosa is also known as gastric siderosis. Gastric siderosis and mucosal ulceration are diagnosed by EGD and biopsy [9]. Common EGD findings include erosion, orange/black mucosal discoloration, and reactive gastropathy [9]. While gross examination of the gastric ulceration may provide evidence of iron-induced injury, biopsy of the gastric ulcer is required to make a confirmatory diagnosis [10]. Marginean et al. theorized three histological patterns (A, B, and C) regarding the cause of gastric siderosis [10]. Pattern A refers to iron deposition in the macrophages, stromal cells, and focally in the epithelium [10]; this pattern is thought to be associated with gastric inflammation, ulceration, prior mucosal hemorrhage, or possibly oral iron medications [10, 11]. Pattern B, also referred to as “iron-pill gastritis,” demonstrates extracellular clumps of crystalline iron deposition with stromal and epithelial deposition and is associated with oral iron medication and mild gastritis [10]. Pattern C has gastric glandular deposition associated with systemic iron overload and hemochromatosis [10]. This patient had mucosal discoloration surrounding the ulcer consistent with iron-associated injury (Figure 1) and a biopsy most consistent with patterns A and C as iron was found within epithelial cells and within glandular cells, suggesting iron overload (Figure 3) despite labs consistent with iron deficiency.

Iron-associated mucosal injury is a well-recognized process in patients with iron overload and tissue deposition secondary to hemochromatosis, oral iron overdose, and frequent blood transfusions [11]. Although the mechanism is not fully understood, it is thought to involve the Fenton reaction, which describes the iron-mediated generation of reactive oxygen species (ROS) [12]. Superoxide ions convert ferric iron (Fe^3+^) to ferrous iron (Fe^2+^) [12]. Ferrous iron reacts with hydrogen peroxide to produce hydroxyl free radicals, which lead to mucosal injury and subsequent inflammation [11, 12]. Additional possibilities include the direct corrosive effect in patients taking oral iron pills but this was not applicable to our patient with no recent oral iron pill therapy [11].

Our patient has a history of compensated cirrhosis secondary to chronic alcohol abuse. Endoscopy also revealed nonbleeding gastric varices which may have also contributed to gastrointestinal bleeding and the patient's need for IV iron and frequent blood transfusions prior to presentation, though no evidence of prior or active bleeding was found. In our patient, his cirrhosis and alcohol abuse, in addition to concomitant IV iron infusions and frequent blood transfusions, may have contributed to gastric siderosis [8, 11, 13]. A study by Hattori reported that 26% of patients with cirrhosis were found to have gastric iron deposition compared to only 4% without cirrhosis [13]. It is possible that cirrhosis can predispose patients to iron-induced gastritis and gastric ulcers if they are receiving additional therapies high in iron concentration, regardless of mode of intake. This case illustrates that patients with underlying risk factors such as alcohol abuse, cirrhosis, and frequent iron intake (regardless of modality) are at increased risk for gastric iron deposition, progression to gastritis and ulceration, and potential upper gastric bleeds. Clinicians should remain vigilant for this possible sequela even if labs do not suggest iron overload. Furthermore, this diagnosis should be on the differential level in patients with treatment-resistant anemia receiving IV iron therapy or frequent transfusions.

## 4. Conclusion

To our knowledge, this is the first reported case of gastric siderosis, gastritis, and gastric ulceration resulting from IV iron and frequent PRBC transfusions. Iron-induced gastritis is a rare, potentially serious, and paradoxical complication of iron supplementation that may not be recognized as a potential cause of refractory iron deficiency anemia. Clinicians should consider iron-induced gastritis in patients with refractory iron deficiency anemia in the setting of frequent iron supplementation and be aware of additional predisposing risk factors such as cirrhosis.

## Abbreviations

WHO: World Health Organization
IDA: Iron deficiency anemia
PRBC: Packed red blood cells
IV: Intravenous
GI: Gastrointestinal
CT: Computed Tomography
EGD: esophagogastroduodenoscopy
PUD: Peptic ulcer disease
H. Pylori: Helicobacter pylori.
